# Disorders of the adrenal cortex: Genetic and molecular aspects

**DOI:** 10.3389/fendo.2022.931389

**Published:** 2022-08-29

**Authors:** Georgia Pitsava, Andrea G. Maria, Fabio R. Faucz

**Affiliations:** ^1^ Division of Intramural Research, Division of Population Health Research, Eunice Kennedy Shriver National Institutes of Child Health and Human Development, National Institutes of Health, Bethesda, MD, United States; ^2^ Section on Endocrinology and Genetics, Eunice Kennedy Shriver National Institute of Child Health and Human Development, National Institutes of Health, Bethesda MD, United States; ^3^ Molecular Genomics Core (MGC), Eunice Kennedy Shriver National Institute of Child Health and Human Development, National Institutes of Health, Bethesda MD, United States

**Keywords:** adrenal cortex, genetics, adrenal tumors, aldosterone secretion, cortisol secretion

## Abstract

Adrenal cortex produces glucocorticoids, mineralocorticoids and adrenal androgens which are essential for life, supporting balance, immune response and sexual maturation. Adrenocortical tumors and hyperplasias are a heterogenous group of adrenal disorders and they can be either sporadic or familial. Adrenocortical cancer is a rare and aggressive malignancy, and it is associated with poor prognosis. With the advance of next-generation sequencing technologies and improvement of genomic data analysis over the past decade, various genetic defects, either from germline or somatic origin, have been unraveled, improving diagnosis and treatment of numerous genetic disorders, including adrenocortical diseases. This review gives an overview of disorders associated with the adrenal cortex, the genetic factors of these disorders and their molecular implications.

## 1. Introduction

Adrenal glands are the major regulators of body homeostasis and endocrine stress response (1). They are small yellowish structures located on the upper poles of the kidneys, in the retroperitoneal area on the lateral edge of the vertebral column. They are found within perirenal fat and are surrounded by the renal fascia. The left adrenal gland is crescent-shaped, while the right is triangular. The weight of each gland in a healthy adult is 8-10 g and the average dimensions are 5.0x3.0x0.6 cm. They are highly vascular and receive their blood supply from 3 arteries: the superior, middle and inferior adrenal arteries. These arise from the inferior phrenic artery, abdominal aorta and renal arteries, respectively (2). The adrenal arteries form a capsular arteriolar plexus, which supplies the adrenal glands. With respect to venous drainage, the right adrenal has a single vein that drains directly to inferior vena cava, whereas the longer left adrenal vein drains into the renal vein (3).

The adrenal glands are comprised of two distinct parts, the cortex and the medulla. The medulla found in the center of the adrenal gland is composed of chromaffin cells and it is dependent on tissue interactions with the adrenal cortex (4). The cortex forms the outer part and is responsible for up to 90% of the adrenal weight. The adult adrenal cortex plays a vital role in normal physiology, being the site of steroid hormone production (3, 5). It consists of three morphologically and functionally distinct compartments. The outer zona glomerulosa (ZG) makes up about 15% of the cortex and produces aldosterone, a mineralocorticoid that controls blood pressure by regulating intravascular volume through sodium and water retention (6, 7). Beneath the ZG, is zona fasciculata (ZF), that comprises a major part of the adrenal gland and is the site of glucocorticoid synthesis. This is where cortisol is produced, a hormone with important effects on the immune system, metabolism and cardiovascular system. The innermost zone is zona reticularis (ZR), which produces adrenal androgens including androstenedione, dehydroepiandrosterone (DHEA), as well as its sulfate, DHEAS (8).

Adrenal cortex cellular function is finely regulated by complex mechanism that involve paracrine and endocrine responses. Dysregulation of signaling pathways in the adrenal cortex is associated with the development of adrenal tumors, some are benign and most rarely, malignant tumors (9).

The advance of new technologies in the field of genetics made possible to determine variations and structures at a genone-wide level (10). Next generation sequencing (NGS) became available at the beginning of the 21st century lowering the costs of DNA sequencing beyond what is possible with standard dye-terminator methods (11). In the clinical context, NGS has greatly improved the discovery of disease associated variants, facilitating not only faster and precise diagnosis but also risk factor prediction for complex disorders (12). For example, a recent study analyzed gene expressions in cortisol-producing adenomas (CPA) with *PRKACA* mutation and compared to *GNAS* and *CTNNB1* mutant CPAs. NGS analysis revealed differences between *PRKACA* mutant and *GNAS* and *CTNNB1* mutant CPAs, such as increased cortisol production in *PRKACA* mutant CPAs (13). This study allows better understanding of pathways involved in CPA and also may direct a more precise treatment approach for those individuals who harbors CPAs. Another study made use of whole-exome sequencing to determine the proportion of cells exhibiting the disease-causing variant *KCNJ5* p.G151R in an individual already diagnosed with bilateral adrenal hyperplasia (BAH). The results indicated a very low-level mosaicism (less than 0.5%) in the germline DNA, while all adrenocortical cells tested from 11 different nodules harbored the disease-causing variant. This finding has implication in patient prognosis and, family risk prediction (14). In this review, we intend to highlight the genomic and molecular aspects of adrenocortical tumors and its implication in patient survival.

## 2. Hormone secretion

The precursor of all adrenal steroid hormones is cholesterol, which is found in circulating low-density lipoprotein (LDL) particles. Briefly, LDL particles are taken up by adrenal cells *via* LDL-receptor mediated endocytosis (15, 16). The vesicles formed during this process subsequently fuse with lysozymes, where hydrolysis generates free cholesterol. Alternatively, cholesterol can either be uptaken from circulating HDL cholesterol *via* the scavenger receptor class B type 1 (SR-B1), or produced de novo from the acetyl coenzyme A (CoA) (17). Cellular cholesterol that is in excess is stored in the form of cholesteryl esters (CEs); the conversion of cholesterol to CEs is catalyzed by the enzyme CoA-acetyltransferase (ACAT) (18). In the adrenal glands, CEs act as the cholesterol ‘storage’ for the production of steroid hormones (18).

### 2.1 Hypothalamic-pituitary-adrenal (Hpa) axis: Glucocorticoid secretion

The secretion of glucocorticoids is regulated by the HPA axis. Their synthesis is stimulated by ACTH, which is released into the bloodstream by the anterior pituitary as part of a 241-amino acid precursor, POMC. In turn, ACTH production is regulated by corticotropin-releasing hormone (CRH), which is released by the neuroendocrine neurons in the paraventricular nucleus of the hypothalamus. Secretion of CRH is dependent on circadian rhythm, as well as various stressors (fever, hypotension, hypoglycemia) acting on the hypothalamus. The HPA axis is a negative feedback system, in which cortisol acts as a direct inhibitor of the synthesis of both ACTH and CRH.

### 2.2 Renin-angiotensin-aldosterone system (RAAS): Mineralocorticoid secretion

Secretion of mineralocorticoids is regulated mainly by the RAAS and potassium, while it also responds acutely to ACTH (19, 20). The juxtaglomerular (JG) cells in the afferent arterioles of the kidney contain prorenin, which is inactive. When JG cells are activated (in response to intravascular volume depletion, or decreased sodium in the distal convoluted tubule or β-activation) prorenin is cleaved to renin (18, 21). Once renin is released in the blood it acts on angiotensinogen, which is synthesized in the liver and is converted to angiotensin I (Ang I) in the kidney by renin. Ang I is then converted to angiotensin II (Ang II) by the angiotensin converting enzyme (ACE) in the lungs. Ang II and potassium increase the expression of aldosterone synthase (*CYP11B2*), while they also stimulate aldosterone production and glomerulosa cell proliferation (22). In turn, aldosterone acts on mineralocorticoid receptors in kidney cells, from the distal convoluted tubule to the cortical collecting tubule. The result of its action is increased sodium reabsorption and excretion of potassium and hydrogen ions.

### 2.3 Adrenal androgen secretion

ZR cells produce androgens, the most important of which are DHEA and DHEAS (23). These are weak precursors that are converted to testosterone and estrogens (such as estradiol) in the peripheral tissues (24). It is established that steroidogenesis is under the control of ACTH which stimulates the transport of intracellular cholesterol into the adrenal cortex (25).

## 3. Disorders of growth of the adrenal cortex

### 3.1 Adrenal hyperplasia

#### 3.1.1 Congenital adrenal hyperplasia (CAH)

CAH is a group of autosomal recessive disorders of the adrenal cortex caused by enzymatic deficiencies in the adrenal steroidogenesis pathway (26, 27). Depending on the degree of residual enzymatic activity, various forms of CAH have been described in the literature, including the most severe form (classic salt-wasting variant), followed by the classic simple virilizing form as well as milder forms (non-classical variant).

##### 3.1.1.1 21OH deficiency

More than 90% of CAH cases are due to deficiency in 21-hydroxylase (*CYP21A2*) (Online Mendelian Inheritance in Man [OMIM] #201910 (28). The gene encoding 21OH, *CYP21A2*, is located on chromosome 6p21.3, within the human leukocyte antigen (HLA) major histocompatibility complex locus (29). *CYP21A2* and *CYP21A1P*, a homologous pseudogene, are approximately 30kb apart. Because of the high degree of sequence homology between these duplicate genes, meiotic recombination events are common in this region. Almost 95% of *CYP21A2* disease causing mutations are *CYP21A1P*-derived variants or deletions used due to recombination events (30, 31). Defects in 21OH result in impaired production of aldosterone and cortisol and elevated precursors, most notably 17-hydroxyprogesterone (17OHP), elevated levels of 17OHP are used for the diagnosis of CAH. In addition, excess of androgens occurs due to constitutive adrenal androgen synthesis, and results in virilization.

The most severe form of 21OH deficiency is due to variants that inactivate *CYP21A2* completely. Without neonatal screening, the phenotype in these cases manifests within the first 2 weeks of life with a life-threatening adrenal crisis (32). In the non-classic cases, the adrenal crisis is prevented. This is because some enzyme activity is preserved, and as a result aldosterone and cortisol production are not completely abolished (28, 33). The non-classic cases are thus characterized by symptoms attributed to the androgen excess: premature puberty, hirsutism and irregular menses. In some cases, patients may present with few or no symptoms and are identified by family genetic studies for other reasons (34). Females with non-classic CAH usually present with similar symptoms as those with polycystic ovary syndrome (PCOS), including hyperandrogenism (clinical or biochemical), and menstrual abnormalities (33, 35–37), and thus is difficult to differentiate between the two, leading to misdiagnosis of non-classic CAH as PCOS in some cases (38–40). Thus, it is suggested that patients undergo measurement of 17OH- progesterone levels followed by ACTH-stimulation test (41, 42).

##### 3.1.1.2 11βOH deficiency

Approximately 8% of CAH cases are due to 11β-hydroxylase (*CYP11B1*) deficiency (43). *CYP11B1*, encoded by *CYP11B1*, is an enzyme regulated by ACTH, which catalyzes the conversion of 11-deoxycortisol to cortisol in the zona fasciculata. Patients with impaired 11-hydroxylation present with decreased corticosterone and cortisol synthesis, accumulation of the precursor deoxycorticosterone, and overproduction of adrenal androgens. Although deoxycorticosterone is a weak mineralocorticoid, in elevated concentrations it mimics the action of aldosterone, suppressing the renin-angiotensin axis, increasing blood pressure, and sometimes causing hypokalemia (43).

##### 3.1.1.3 17OH deficiency

Deficiency of 17α-hydroxylase (*CYP17A1*) is rare, and severely damaging variants in *CYP17A1* result in absent cortisol as well as androgens, causing puberty failure and sexual infantilism (44). *CYP17A1* is expressed in the ZF and the ZR, but not in the ZG. Both 46,XY and 46,XX patients with 17OH deficiency have female external genitalia, and present at puberty as phenotypically female. They have hypergonadotropic hypogonadism without secondary sexual characteristics, and low-renin hypertension.

##### 3.1.1.4 *3βHSD2* deficiency

There exist two isoforms of 3β-hydroxysteroid dehydrogenase: 3βHSD1 and *3βHSD2*, encoded by *HSD3B1* (the homologous type I gene) and *HSD3B2*, respectively. *HSD3B1* is expressed in the placental and peripheral tissues (breast, prostate and skin), while *HSD3B2* is expressed exclusively in the adrenals and gonads (45). *3βHSD2* deficiency is characterized by deficiency of both glucocorticoids and mineralocorticoids, as well as by dehydroepiandrosterone (DHEA) overproduction. DHEA is converted to testosterone by extra-adrenal 3βHSD1, and patients present in infancy with underdeveloped 46,XY genitalia and – rarely – 46,XX virilization (46).

##### 3.1.1.5 Lipoid congenital adrenal hyperplasia

The most severe defect of steroidogenesis is lipoid congenital adrenal hyperplasia (LCAH). LCAH is caused by defects in the steroidogenic acute regulatory protein (StAR) and is characterized by deficiency of all steroid hormones. StAR regulates the transfer of cholesterol from the outer to the inner mitochondrial membrane, a vital step in the initiation of steroidogenesis. As a result, cholesterol cannot be mobilized. Adrenal lipid droplets subsequently accumulate and are seen on the autopsy, thus the name of the disorder. In both 46,XY and 46,XX patients, it presents with female external genitalia and an adrenal crisis in the neonatal period (47).

Regarding the current treatment for CAH, there is no consensus yet, therefore, it still remains a challenge. It usually includes glucocorticoid and mineralocorticoid replacement therapy (48).

### 3.2. Adrenocortical tumors

Adrenocortical tumors (ACTs) can be sporadic or familial, unilateral or bilateral, and non-secreting or secreting. The latter secretes various adrenal steroid hormones; the exact hormone varies depending on the tumor type. Unilateral ACTs are common, and approximately 10% of the general population appears to have an adrenal cortical lesion (49). They are often discovered incidentally when evaluating for another disease and are thus called incidentalomas (50, 51). Once discovered, they are evaluated by abdominal computed tomography (CT). The vast majority of them are benign adrenocortical adenomas (ACAs). Some ACAs are non-secreting, while others can secrete cortisol and cause Cushing syndrome (5-47% of cases), or aldosterone and cause Conn syndrome (1.6-3.3%) (50, 52). The rest of ACTs are adrenocortical carcinomas (ACCs), which are rare (prevalence 4-12 cases per million).

#### 3.2.1 Benign cortisol-producing adrenocortical tumors

Cushing’s syndrome (CS) has an estimated incidence of 39-79 per million people per year in various populations, with a female-to-male ratio of 3:1 (53–56). Data from various studies suggest that there is an increased prevalence in people with early-onset osteoporosis, type 2 diabetes, or hypertension, but the precise estimates vary (57–60).

CS is characterized by cortisol overproduction. The cause of 80% of endogenous CS cases is over-secretion of ACTH by a pituitary corticotroph adenoma or – less frequently – by a neuroendocrine tumor (61–63). In rare cases, neuroendocrine tumors such as pheochromocytoma and medullary thyroid carcinoma produce corticotropin-releasing hormone (CRH), which then results in pituitary ACTH over-secretion (61–63). In 20% of the cases, CS is ACTH-independent, and the cause is the primary overproduction of cortisol by the adrenal glands. In such cases, the most frequent underlying pathology is a cortisol-producing adenoma, while adrenocortical carcinomas and bilateral adrenal hyperplasia are responsible for less than10% of the cases (64). Bilateral adrenal hyperplasia in particular may be either isolated, or part of a syndrome, and can be divided into two entities based on the size of the nodules: primary bilateral macronodular adrenal hyperplasia (PBMAH), which is characterized by several nodules (diameter >10mm) (65), and two micronodular forms. The latter are primary pigmented micronodular adrenal hyperplasia (PPNAD) and isolated micronodular adrenocortical disease (iMAD) (diameter <10mm) (61–63, 66).

**Figure 1:**
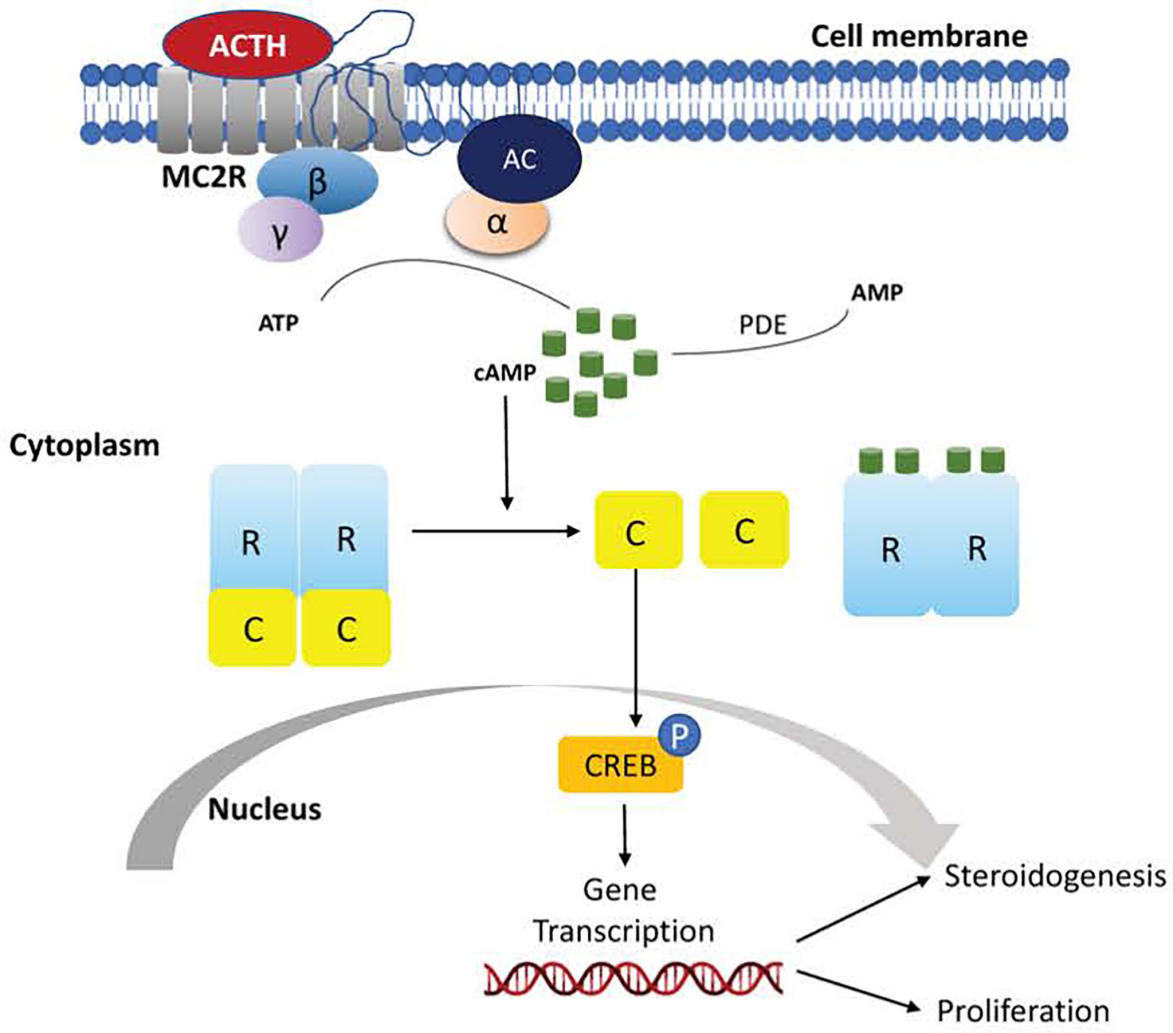
Schematic representation of activation of the cyclic adenosine monophosphate (cAMP) signaling pathway in normal adrenocortical cells.

The cAMP/PKA pathway is the main regulator of cortisol production (67). PKA (protein kinase A) consists of two regulatory subunits and two catalytic subunits that – under normal conditions – are bound together. In adrenocortical cells, the pathway is activated by the binding of ACTH to *MC2R*, a G protein-coupled receptor (GPCR). This triggers an increase in cAMP levels, which binds to the PKA regulatory subunits causing the release from the catalytic subunits. The catalytic subunits, then translocate to the nucleus, where they phosphorylate, and thus activate, transcription factors that promote cortisol synthesis (**Figure 1**) (68).

#### 3.2.2 Primary bilateral macronodular adrenal hyperplasia (PBMAH)

PBMAH is usually diagnosed in patients at 40-65 years old that present CS and low levels of ACTH, or - more recently - when investigating an adrenal incidentaloma. Many terms have been used over the years to describe PBMAH. Such terms include primary macronodular adrenal hyperplasia (PMAH), autonomous macronodular adrenal hyperplasia (AMAH), bilateral macronodular adrenal hyperplasia (BMAH), and ‘huge’ or ‘giant’ macronodular adrenal disease. Another term, ACTH-independent massive bilateral adrenal disease (AIMBAD), has also been used in the past, but in later studies the secretion of cortisol appeared to be regulated by corticotropin and thus, this term is not used anymore (69).

In general, PBMAH presents with bilateral macronodules and enlargement of the adrenal glands. In the majority of cases (77-87%), the macronodules exhibit ectopic or excessive expression of G-protein coupled receptors, including luteinizing hormone/choriogonadotropin (LH/hCG) responsible for Cushing syndrome during pregnancy and after menopause (70), glucose-dependent insulinotropic peptide (GIP) that is responsible for food-dependent Cushing syndrome (71), serotonin 5HT, catecholamines, Ang II, glucagon and vasopressin (65, 72–77). The binding of these receptors to their ligands mimics the result of ACTH binding to *MC2R* leading to activation of cAMP/PKA pathway and thus excessive cortisol secretion (78). So far, the exact molecular mechanism of the ectopic receptor expression has not been completely elucidated (79).

Genetic variants resulting in increased activity of the cAMP/PKA pathway *via* a variety of mechanisms have been reported in patients with PBMAH. For example, variants in *PDE11A*, that encodes phosphodiesterase type 11A, have a prevalence of 24-28%, whereas inactivating germline variants in *PDE8B*, encoding phosphodiesterase type 8B, and *PRKACA* copy number gains, have also been encountered (80–83). Another component of the cAMP/PKA pathway associated with PBMAH is the Gα subunit, encoded by **GNAS*1* (84). Activating variants in **GNAS*1* cause McCune-Albright syndrome, which is associated with ‘café au lait’ spots, polyostotic fibrous dysplasia, precocious puberty and hyperfunction of multiple endocrine glands (84). These **GNAS*1* variants are somatic and lead to continuous activation of the cAMP/PKA pathway and thus, cortisol-producing adenomas (85). Finally, in a case report of an isolated case of bilateral adrenal hyperplasia, the synergistic action of two variants (p.C21R and p.S247G) on the same allele of *MC2R* (encoding the melanocortin 2 receptor/ACTH receptor) resulted in autonomous cortisol secretion *via* constitutive activation of the cAMP/PKA pathway (86). This case is remarkable in that if those defects had happened in isolation, they would have led to receptor inactivation (86).

Rarely, PBMAH can occur as part of genetic tumor predisposition syndromes such as familial adenomatous polyposis (*APC*), multiple endocrine neoplasia type 1 (*MEN1*) and hereditary leiomyomatosis (*FH*) (83, 87–89). It is important to mention that these genetic alterations are associated with other tumors and are responsible only for a limited number of PBMAH cases. Additionally, PBMAH can be associated with aromatase expression leading to elevated estrogens, independently of sex (79).

More recently, germline variants in the armadillo repeat-containing 5 (*ARMC5*) have been found to implicate in the pathogenesis of PBMAH, with their prevalence estimated between 21%-26% (90, 91). *ARMC5* is a cytosolic protein that had no enzymatic activity, and its function depends on interactions with other proteins (92). *ARMC5* is located on chromosome 16p11.2. In mice, as well as *in vitro*, *ARMC5* has been shown to play an important role in regulating steroidogenesis, proliferation, apoptosis, T-cell differentiation and immune responses (92–95). Most of the patients harboring *ARMC5* variants had adrenal CS, with the hypersecretion of cortisol being more severe compared to that seen in patients that had *ARMC5* variants that were predicted to be benign or did not have *ARMC5* variants. In addition, damaging variants or deletions in *ARMC5* were identified in several families with PBMAH (96, 97), whereas an association of *ARMC5* with primary hyperaldosteronism was also reported in 2015 (98).

In a small number of cases, somatic variants in genes participating in other biological processes have been described. These include two chromatin regulator genes, *DOTIL* (that encodes a histone H3 lysine methyl-transferase) and *HDAC9* (that encodes a histone deacetylase) (99). In addition, a study of two siblings from a family that segregated PBMAH implicated the Endothelin Receptor type A *EDNRA*, which encodes a G-coupled protein. However, this association remains to be confirmed in follow-up studies (100).

A meaningful update of the 2022 WHO classification of adrenocortical tumors was recently summarized by Mete and co-workers early this year. As a result of the advance on next generation sequencing studies, it is possible to recognize that PBMAH is caused by germline variants in one out of many susceptible genes, with a second hit in the somatic cells. These findings strongly suggest a neoplastic instead of hyperplastic condition. To avoid a misnomer for the disease, the 2022 WHO classification changed the nomenclature of primary bilateral macronodular adrenal hyperplasia to bilateral macronodular adrenocortical disease (101).

#### 3.2.3 Primary pigmented nodular adrenal disease (PPNAD)

PPNAD is most commonly diagnosed in children and young adults and is a rare cause of ACTH-independent hypercortisolism. It is most commonly part of Carney complex (CNC), an autosomal dominant tumor predisposition syndrome. CNC presents with various endocrine tumors including pituitary adenomas, thyroid benign tumors, and testicular Sertoli cell-calcified tumors, as well as non-endocrine tumors, most commonly pigmented skin lesions, skin and cardiac myxomas (102, 103). PPNAD in patients with CNC has a prevalence close to 60% (104, 105).

With respect to the molecular background, CNC is caused by germline inactivating variants in the PRKAR1A gene, located at the 17q24.2-24.3 locus (CNC1 locus). PRKAR1A variants are found in 37% of patients with the sporadic form of the disease and in more than 70% in the typical familial forms, with almost 100% penetrance (105, 106). PRKAR1A encodes the regulatory subunit type 1α (R1α) of PKA. As a result, inactivating PRKAR1A variants result in aberrant activation of the cAMP/PKA pathway. To date, approximately 140 pathogenic variants have been reported (https://PRKAR1A.nichd.nih.gov/hmdb/mutations.html). The majority of them are located in exons 2,3,5,7, and 8 while about 20% are located in intronic sequences and affect splicing (105, 106). Most of the variants are small deletions and insertions, base substitutions or combined rearrangements involving up to 15bp (107). In almost all cases (90%), the genetic alteration leads to a premature stop codon. Subsequently, the transcripts containing the premature stop codon are degraded by nonsense mediated decay (NMD). As a result, the amount of RIα protein produced is half of the normal amount (107–109). Large chromosomal deletions involving the PRKAR1A gene, even though rare, have also been identified (110, 111). Occasionally, the pathogenic variant (missense, short in-frame insertions/deletions and splice variants) may lead to the production of an abnormal protein that is incapable of responding appropriately to cAMP levels or properly bind to the PKA catalytic subunits (108, 112).

In some families without variants in PRKAR1A, the causative gene has not been identified yet; however, genetic linkage analysis of tumors has shown that there is another affected locus on chromosome 2p16 (CNC2 locus) (113, 114). The majority of those cases have been diagnosed with CNC later in life (115). In addition, a single patient with CNC that presented with abnormal skin pigmentation, acromegaly and myxomas, was found to harbor copy number gains of locus containing the *PRKACB* gene (116). Moreover, a recent study with 353 CNC patients and/or PPNAD, showed that the majority of patients with isolated PPNAD harbored a germline c.709-7del6 variant (105).

During the past years, variants affecting the phosphodiesterase genes *PDE11A* and *PDE8B* have emerged as putative causes of PPNAD. The loci harboring these genes had the most significant associations in a genome-wide association study performed on individuals lacking genetic defects in *GNAS* or PRKAR1A, while the locus harboring *PDE11A* showed the largest loss-of-heterozygosity in tumor samples (117). In addition, targeted sequencing of *PDE11A* revealed that patients with CNC that also had PPNAD and/or testicular large cell calcifying Sertoli cell tumors were more likely to have variants in *PDE11A*, compared to patients without these tumors (118). All of these patients also had germline variants in PRKAR1A, raising the possibility that *PDE11A* variants act as genetic modifiers that elevate the risk for PPNAD and/or LCCSCT in CNC. With regards to *PDE8B*, a single nucleotide variant (c.914A>C/p.His305Pro) was detected in a 2 year old female patient diagnosed with PPNAD; the variant was inherited from the patient’s father. This variant was subsequently shown to lead to decreased *PDE8B* activity *in vitro* (119).

It is worth noting that variants in *PDE11A* and *PDE8B* have been found in other types of adrenocortical tumors as well. In addition to PBMAH tumors (described above), haploinsufficiency of *PDE11A* has been implicated in ACA and ACC (80), and in vitro studies have demonstrated that putative PBMAH-causing variants compromised the enzymatic activity of *PDE11A* (81). Furthermore, in a cohort of patients with adrenocortical tumors without variants in PRKAR1A, *GNAS*, or *PDE11A*, 7 patients harbored variants in *PDE8B* (82). Two of these variants were then experimentally shown to decrease protein activity (82).

Recently, genes encoding for the catalytic subunits of PKA have also been implicated in micronodular PBMAH. A study of two patients with familial PBMAH, and of three patients with sporadic iMAD, identified germline copy number gains of the locus that harbors *PRKACA*, which encodes the Ca catalytic subunit. Tumor samples from these patients revealed elevated basal as well as cAMP-stimulated PKA activity (120, 121). In addition, copy number gains involving the *PRKACB* locus (which encodes the Cβ catalytic subunit), were reported in a patient with CNC that presented with myxomas, acromegaly, and abnormal skin pigmentation. The patient was found to have increased cAMP-induced kinase activity in lymphocytes, resembling what is seen in CNC patients with PRKAR1A variants. Moreover, increased Cβ levels were found in several cell types as well as in breast myxoma cells (116).

Finally, the wingless-type-(Wnt)-β-catenin pathway has also been suggested to play a role in micronodular BAH. Somatic variants in the β -catenin gene (*CTNNB1*) were found in two (11%) patients with PPNAD in a previous study, with one of these patients also harboring a PRKAR1A variant. These variants were encountered in larger adrenocortical adenomas that arose within the context of PPNAD, and were absent from the surrounding hyperplastic adrenocortical tissue (122). A different study landed further support to the involvement of the Wnt-β-catenin pathway, by showing accumulation of β-catenin in PPNAD tissues, as well as activating somatic *CTNNB1* variants in macronodules, but not in micronodules or the contralateral adrenal gland (123).

#### 3.2.4 Cortisol-producing adrenocortical adenomas (ACA)

Cortisol-producing adenomas exhibit overactivation of the cAMP/PKA signaling pathway. The most prevalent CPA-causing defect is alteration of the catalytic α-subunit of PKA (*PRKACA*) and the most common variant has been reported to be p.Leu206Arg (124). Other rare variants have been described (120, 125, 126), all localized in a region of *PRKACA* that affects its interaction with the regulatory subunit 1α. Activating variants in *PRKACA* lead to continuous activation of PKA by abolishing the interaction between its catalytic and regulatory subunits. In addition, they can lead to hyperphosphorylation of certain substrates, thereby altering substrate specificity (127). An activating somatic variant in *PRKACB* has also recently been reported in a patient with CPA; *in vitro* studies showed that this variant confers higher sensitivity to cAMP (128).

Furthermore, somatic inactivating variants in *GNAS* and PRKAR1A have been found in sporadic adrenocortical tumors (126, 129–133). The genetic alterations in both genes lead to increased signaling *via* the cAMP/PKA pathway, however they activate different downstream effectors. Adrenal lesions that harbored variants in *GNAS* or PRKAR1A had overactivation of the p53 and MAPK signaling pathways, respectively. In PRKAR1A-mutant tumors, genes related to Wnt-signaling pathway (*CCND1*, *CTNNB1*, *LEF1*, *LRP5*, *WISP1* and *WNT3*) were overexpressed, while in *GNAS*-mutant tumors, there was increased expression of extracellular matrix receptor interaction and focal adhesion pathways (NFKB, *NFKBIA* and *TNFRSF1A*) (134).

#### 3.2.5. Aldosterone-producing benign adrenocortical neoplasms

Excess of aldosterone production characterizes a group of adrenal cortical disorders including aldosterone-producing adenomas, adrenal cortical hyperplasia, familial hyperaldosteronism (*FH*, <1%) and rarely, carcinomas (<1%) (135–137).

#### 3.2.6 Benign adrenocortical tumors producing aldosterone

Primary aldosteronism (PA) is the most frequent secondary form of hypertension, accounting for approximately 10-20% of patients referred with resistant hypertension and 5% of patients in primary care (138, 139). PA is typically due to unilateral adenomas that produce aldosterone (APA) (65%), or BAH (35%) that leads to autonomous aldosterone production (140). The remaining cases include unilateral hyperplasia (2%), familial hyperaldosteronism (*FH*, <1%) and aldosterone-producing ACC (<1%) (137). The vast majority of PA cases are sporadic and only 6% are familial (141).

##### 3.2.6.1 Inherited forms of PA

Four forms of *FH* (type I-type IV) have been described so far and they are inherited in an autosomal dominant manner (136).

The underlying cause of *FH* I (also known as glucocorticoid-remediable aldosteronism-GRA) is the formation of a chimeric gene, resulting from an unequal fusion of the regulatory regions of *CYP11B1*, which encodes 11β-hydroxylase, and *CYP11B2*, that encodes aldosterone synthase. Both enzymes are responsible for the last steps of cortisol and aldosterone synthesis, respectively (142, 143). Formation of the chimeric gene leads to aldosterone overproduction under the regulation of ACTH (143). Treatment is based on the use of glucocorticoids (144). Genetic testing for the chimeric gene (*CYP11B1*/*CYP11B2*) should be considered for patients who are diagnosed with PA and have a family history of the disease, onset of PA before the age of 20 years, or family history of stroke at a young age (145).

*FH* II is due to mutations in the *CLCN2* gene, which encodes the chloride channel CIC2. Among other tissues, CIC2 is expressed in the adrenal glands. Gain-of-function variants in *CLCN2* lead to increased Cl− ions efflux, which causes cell membrane depolarization and opening of voltage-gated calcium channels, triggering aldosterone production (146, 147). *FH* II is the most common form of *FH*, with a prevalence of 1.2-6% in patients with PA (141, 148, 149).

*FH* III is due to coding variants in the G-protein coupled inward rectifying potassium channel 4 (GIRK4), which is encoded by *KCNJ5*. Genetic defects in this gene cause a lack of ion selectivity and increased sodium influx, which results in cell depolarization triggering calcium entry into the cells. This signals an increase in *CYP11B2* expression and increase in aldosterone production. The severity of hyperaldosteronism has been shown to be related to the type of *KCNJ5* variant in some patients (150–152), but not in all of them (153). The majority of patients with germline variants in *KCNJ5* present with polydipsia, polyuria and refractory hypertension during childhood. In these patients, aldosterone hypersecretion is high enough to require bilateral adrenalectomy (145). There is heterogeneity in the age at which patients present the disease, and in some cases, the symptoms can be controlled with mineralocorticoid-receptor antagonists (MRAs) (150–154).

*FH* IV is caused by germline variants in *CACNA1H*, which encodes the pore-forming α1 subunit of the T-type calcium channel Cav3.2. These variants cause alterations in calcium current properties, leading to increased intracellular calcium concentration and production of aldosterone. Germline variants in *CACNA1D*, which encodes Cav1.3 (the α1D subunit of the voltage-dependent L-type calcium channel) have also been described in patients with PA; these variants occur exclusively de novo. These patients present with a severe early-onset form of hyperaldosteronism associated with a complex neurological disorder, with the phenotype also including seizures and neurological abnormalities (PASNA) (155).

Finally, germline variants in *PDE2A*, *PDE3B* and *ARMC5* have also been reported in patients with PA (98, 156). The first two genes were associated with PA within the context of BAH; however, these are not considered as genetic causes of *FH* (156).

##### 3.2.6.2 Aldosterone-producing adrenocortical adenomas (APA)

In the past decade, major advances have been made in unraveling the molecular background of APAs (157, 158). Variants have been identified in genes associated with familial forms of APA, including *KCNJ5* and *CACNA1D* as well as in *ATP1A1* and *ATP2B3* (which encode two Na+/K+ and Ca2+ ATPases) (155, 159–161). The most frequent defects are recurrent variants in *KCNJ5*, encountered in more than 40% of APAs in Caucasians, with two particular variants (p.G151R and p.L168R) being responsible for 36% of cases (162). Variants in *KCNJ5* appear to be more frequent in Asian cohorts (162–166) (~70% prevalence), and in women compared to men (63% vs 24% prevalence) (163). Variants in *CACNA1D* are reported in up to 10% of patients with APA, while *ATP1A1* and *ATP2B3* variants are less frequent (167).

Additionally, the Wnt/β-catenin signaling pathway has been shown to play a vital role in the adrenal cortex development and the biosynthesis of aldosterone (168). It has been shown to be continuously active in approximately 70% of APAs (169). Under normal unstimulated conditions, β-catenin is in the cytosol as part of the axin complex along with Caseine Kinase 1β, glycogen synthase kinase 3β and adenomatous polyposis coli (*APC*). Binding of the Wnt ligand to its receptor causes β-catenin to dissociate from the axin complex and translocate to the nucleus where it induces the expression of the transcription factors T cell factor (TCF) and lymphocyte enhancer factor (LEF) (168). Somatic variants in *CTNNB1* gene, encoding β-catenin, have been identified in 2-5% of patients with sporadic APA (155, 170, 171); similarly, to APAs due to *KCNJ5* variants, these APAs are associated with larger adenomas and are more commonly seen in females. Variants in *CTNNB1* have also been reported in two pregnant patients with APA that exhibited increased adrenocortical expression of the gonadotropin releasing hormone (GnRH) and LH/hCG receptors (172). Based on this, an association with pregnancy or menopause was suggested, but this was not confirmed in a follow-up study (172). In rare cases, somatic variants in *PRKACA* and *GNAS* have been described in patients with cortisol and aldosterone co-secreting adenoma as well (173, 174). However, their role in the development of APA remains unclear, because those variants are similar to the ones found in CPAs and ACC (99, 120, 131, 175–177).

### 3.3 Adrenocortical carcinoma

ACCs are rare tumors derived from the adrenal cortex. They affect both adults and children with an annual incidence of 0.7-2.0 cases per million per year (178, 179). They are responsible for steroid excess in 60-70% of cases (52, 180, 181) and they represent one of the most aggressive class of endocrine tumors with an overall poor prognosis (5-year survival rate <35%) (129). However, the exact 5-year survival rate varies depending on the tumor stage, from 82% for tumors in stage I to 18% for tumors in stage IV (182). Thus, the stage at the time of the diagnosis is a crucial prognostic factor. Approximately 40-60% of patients present with signs and symptoms related to hormone excess (183–185). Another 30-40% present with non-specific symptoms associated with local tumor growth (30-40%), including early satiety, abdominal fullness and flank or abdominal pain (184, 185). The remaining 20-30% of ACCs are discovered incidentally on imaging studies for unrelated medical conditions (**Table 1**) (187). With regards to the age distribution, ACC seems relatively more common in children than in adults and is often associated with hereditary tumor syndromes (186, 188). In fact, the elucidation of genetic alterations underlying familial syndromes predisposing to ACC has led to the identification of signaling pathways involved in the development of cancer such as Insulin growth factor 2 (IGF-2), Wnt-beta catenin and p53 pathways (189).

**Table 1:** Hormone secretion of functional adrenocortical carcinomas.

Hormone	Symptoms	Incidence
**Cortisol** (186)	Osteoporosis, early onset hypertension, hyperglycemia/diabetes, facial plethora, muscle weakness	50-80%
**Androgen** (49, 186)	Acne, hirsutism, menstrual abnormalities, male baldness	40-60%
**Estrogen** (186)	Testicular atrophy, gynecomastia	1-3%
**Aldosterone** (157)	Hypertension, muscle weakness	Rare

#### 3.3.1 Molecular basis of ACC: As part of a tumor predisposition syndrome

##### 3.3.1.1 Li-Fraumeni syndrome (LFS)

LFS is an autosomal dominant disorder that predisposes to various types of cancer including brain cancer, leukemia, soft tissue sarcoma and osteosarcoma, premenopausal breast cancer and ACC. LFS accounts for 50-80% of pediatric cases of ACC (190–192). The clinical criteria for the diagnosis of ‘classic’ LFS include a sarcoma diagnosis before the age of 45, with a first-degree relative diagnosed with any type of cancer before 45 years and another first or second-degree relative with any cancer diagnosis before the age of 45 years or a sarcoma diagnosis at any age (193). Germline variants in *TP53*, the underlying genetic cause of LFS, have been identified in 70% of cases, while de novo variants have been shown to have a prevalence of 7-20% (129, 190, 194). In a cohort of 286 *TP53*+ patients from 107 families, the cumulative cancer incidence was 50% by 31 years for females and by 46 years for males (190). Of those patients, 67% had their first cancer diagnosis before the age of 17 years, while five patients were diagnosed with ACC before the age of 17 years. Of those patients, 50% had a second cancer diagnosis and ACC was present in one of them (190).

##### 3.3.1.2 Beckwith-Wiedemann syndrome (BWS)

BWS is a systemic overgrowth disorder caused by genetic or epigenetic changes that ultimately result in upregulation of insulin-like growth factor 2 (*IGF2*) (195). Loss of heterozygosity of the 11p15 locus, which harbors *IGF2*, is a common finding in childhood ACC (196). BWS is characterized by hemihypertrophy, macrosomia, macroglossia, hyperinsulinism, omphalocele and distinct facial features (197). In addition, in the first 8 years of life, patients with BWS are at increased risk for embryonal tumors including hepatoblastoma, neuroblastoma and Wilms’s tumor (197–201). The risk of developing intra-abdominal tumors is approximately 5-10% and thus patients with BWS need to undergo regular screening for early diagnosis and management. ACC is the next most common type of tumor reported in BWS patients; other common benign adrenal pathologies include adrenal cysts and ACAs (200).

##### 3.3.1.3 Multiple endocrine neoplasia 1 (*MEN1*)

*MEN1* is inherited in an autosomal dominant manner and is caused by germline heterozygous variants in the *MEN1* gene on chromosome 11q13. Its main manifestations are hyperparathyroidism (95%), entero-pancreatic neuroendocrine tumors (50%) and pituitary adenomas (40%). Associated adrenal lesions, mostly ACA and hyperplasias, are present in 20-55% of *MEN1* cases (202). Most of these adrenal lesions are nonfunctional (87, 203, 204). ACC occurs only in a small fraction of patients with *MEN1* (87, 205, 206). In two cohorts of sporadic ACC, somatic variants of *MEN1* were shown to have a prevalence of 7% (207, 208).

##### 3.3.1.4 Lynch syndrome

ACC has also been reported in cases of Lynch syndrome (hereditary nonpolyposis colorectal cancer, HNPCC) (209–213). Lynch syndrome is an autosomal dominant disorder caused by germline heterozygous coding variants in DNA-mismatch repair genes (*MSH2*, *MSH6*, *MLH1* and PMS2). Patients have a significantly increased risk of cancer, especially colorectal and endometrial cancer, and thus screening for Lynch syndrome is recommended in all patients that are diagnosed with colorectal cancer (209, 214). The prevalence of Lynch syndrome in a large cohort of patients with ACC was reported to be approximately 3% (215).

#### 3.3.2 Other

ACC has been reported in patients with neurofibromatosis type 1, familial adenomatous polyposis, and Werner syndrome (186, 216–223). In addition, ACC has been reported in two cases of patients with CNC (224, 225).

In general, the discovery of genetic syndromes that confer an increased risk for ACC has yielded important clues into the molecular basis of ACC development. For example, the association between FAP and adrenal tumors provided the basis for the insights into the role of β-catenin signaling in adrenal tumors, while the link between ACC and BWS combined with gene expression profiling suggested the IGF-1 receptor as a target for ACC therapy. The latter hypothesis has now been tested in clinical trials (226).

## 4. Treatment approaches and clinical trials

Patients with unilateral adrenal adenomas that are hormonally active receive treatment with unilateral adrenalectomy (25). Individuals diagnosed with CS are evaluated for the need of adrenalectomy based on the degree of cortisol excess, comorbidities, age and preference of the patient; typically, surgical resection of the is the first-line treatment (50). However, in some cases where hypercortisolemia is resistant to surgery or in cases where surgical treatment is contraindicated due to patient co-morbidities pharmacotherapy is indicated (227). This includes ketoconazole along with metyrapone, mitotane, or etomidate (227, 228). Recently, two new drugs were approved by the FDA, levoketoconazole and osilodrostat, which are both inhibitors of steroidogenic enzyme activity. Non-functioning adrenal tumors are evaluated to verify eligibility for partial adrenalectomy based on size and potential to malignancy (229). In general, surgery should be considered in lesions >4cm in size or those that are hormonally indeterminate, even if imaging characteristics resemble those of a benign lesion (50). Based on the guidelines from the Endocrine Society, the diagnosis of PA in patients with hypertension should include screening (elevated aldosterone-renin ratio) followed by confirmatory testing (including salt loading, fludrocortisone or captopril administration, which all fail to sufficiently lower aldosterone levels in patients with PA) (140). Regarding ACCs, complete surgical resection remains the only curative treatment in patients with resectable stages I-III (187, 230), while adjuvant therapies are used to decrease recurrence. In addition, Mitotane, a dichlorodiphenyltrichloroethane analog (DTT), is the only medication that is specifically approved for ACC and can be used either as adjuvant or in advanced stages in combination with classic cytotoxic agents (231). Phase I trial studies using cixutumumab, an insulin growth factor receptor (IGF-1R) antibody have showed promising results in terms of lower toxicity and better disease outcome compared to mitotane (232). Moreover, a trial investigating the combination of cixutumumab with the mTOR inhibitor, temsirolimus, showed that almost half of the patients achieved prolonged stable disease and therefore, current treatment options may be improved (233).

There are at least 15 clinical trials currently recruiting patients diagnosed with ACC. Location includes United States, Europe and China. Over 20 studies, all over the world, are recruiting patients diagnosed with hyperaldosteronism, comprising either observational or interventional studies. There are also about 20 studies currently recruiting patients with CS and many other studies are now recruiting for other adrenal cortex-related diseases. Information about current and past trials, as well as institution and collaborators, can be found at clinicaltrials.gov, a resource provided by the United States National Library of Medicine.

## 5. Conclusion

Here, we summarized the main genetic and molecular aspects of adrenocortical diseases, which are, in some cases, difficult to diagnose. The rapid progress of next generation sequencing (NGS) techniques has opened new horizons to examine and diagnose adrenocortical diseases with unbiased mechanisms. For example, exome sequencing may be used in patients with a clinical phenotype but no identified variant in one of the known causative genes, in order to perform an unbiased scan and potentially identify causative variants in genes previously not associated with the disorders. This not only yields a diagnosis, but also provides new clues into disease pathophysiology. Despite the progress, diagnosis based on genetic screening is still limited to either large centers and/or patients with financial access to these analyses. Therefore, heath care providers still face limitations in offering access to precise medicine to all patients. Considering that a great number of adrenocortical diseases is due to genetic onset, we believe that increasing access to NGS will greatly improve early and precise diagnosis of adrenocortical disorders. Consequently, more accurate treatments will be delivered to individuals that harbor genetic alterations leading to disorders of the adrenal cortex.

## Author contributions

GP: Writing - original draft; AM: Writing - review & editing; FF: Conceptualization; Supervision; Writing - review & editing. All authors contributed to the article and approved the submitted version.

## Conflict of interest

The authors declare that the research was conducted in the absence of any commercial or financial relationships that could be construed as a potential conflict of interest.

## Publisher’s note

All claims expressed in this article are solely those of the authors and do not necessarily represent those of their affiliated organizations, or those of the publisher, the editors and the reviewers. Any product that may be evaluated in this article, or claim that may be made by its manufacturer, is not guaranteed or endorsed by the publisher.
